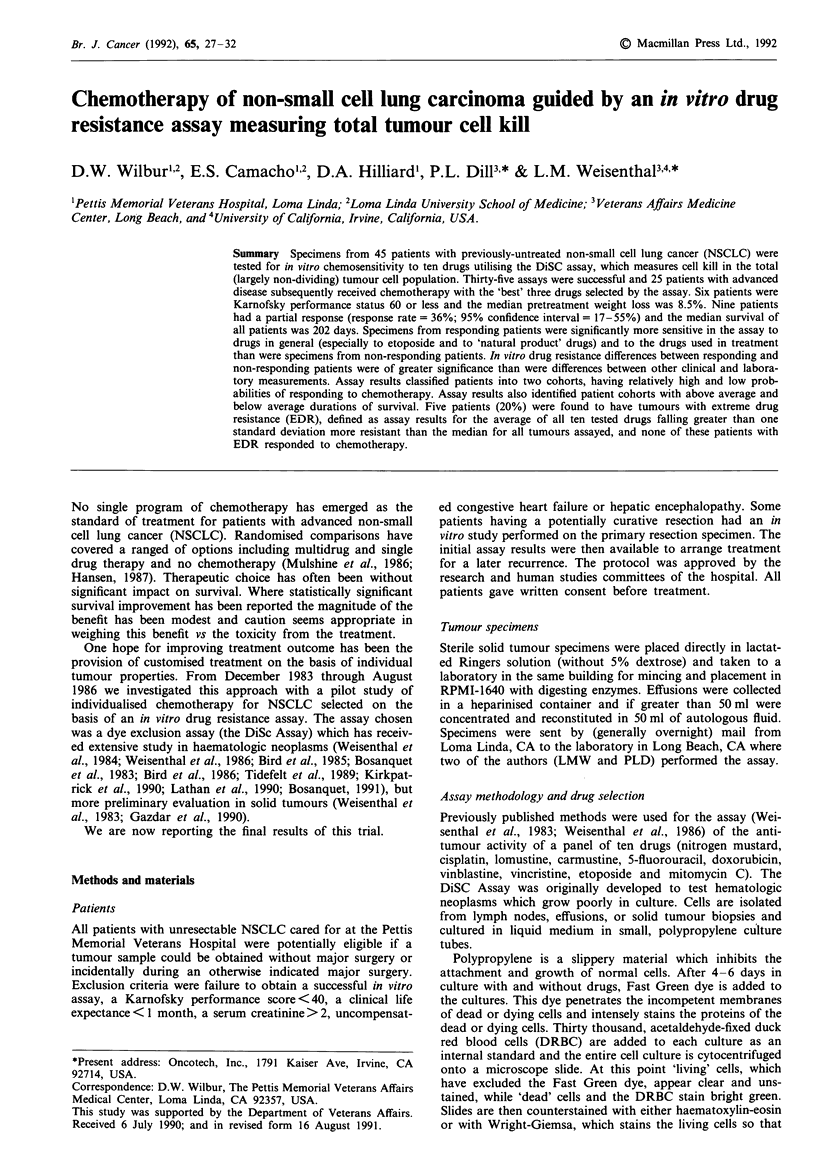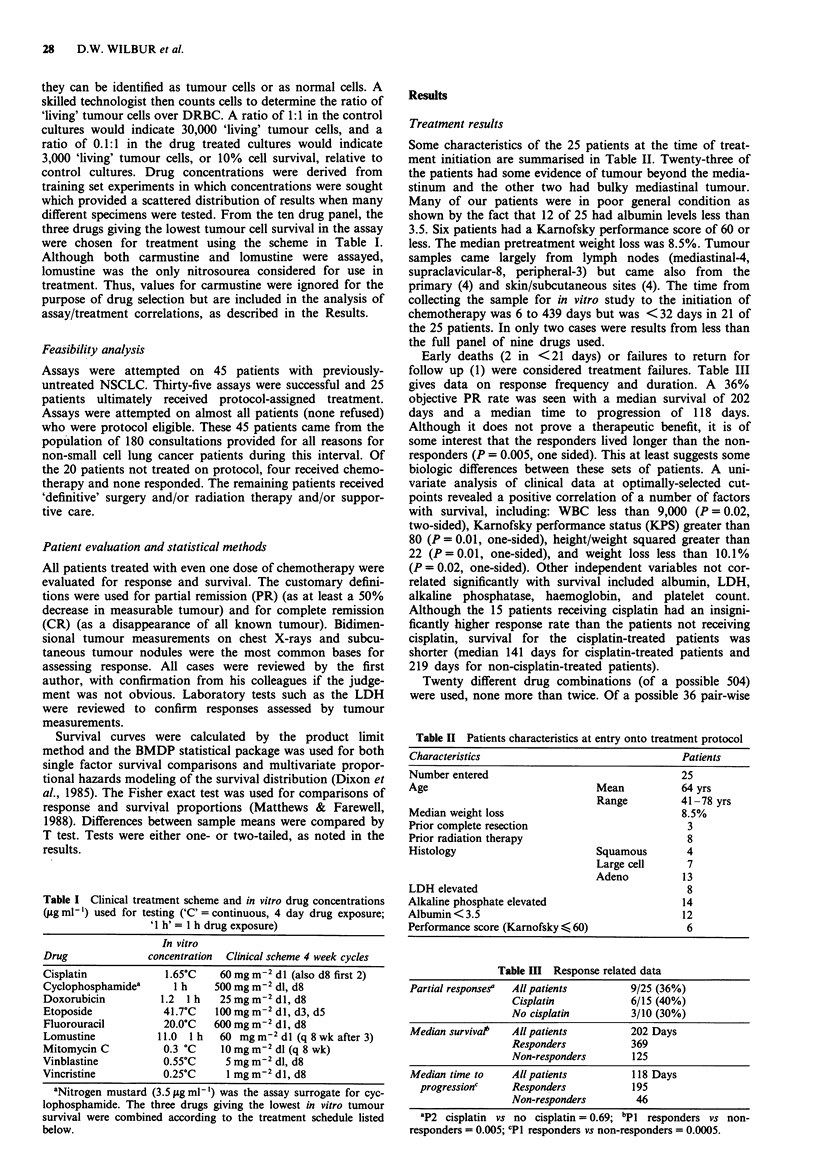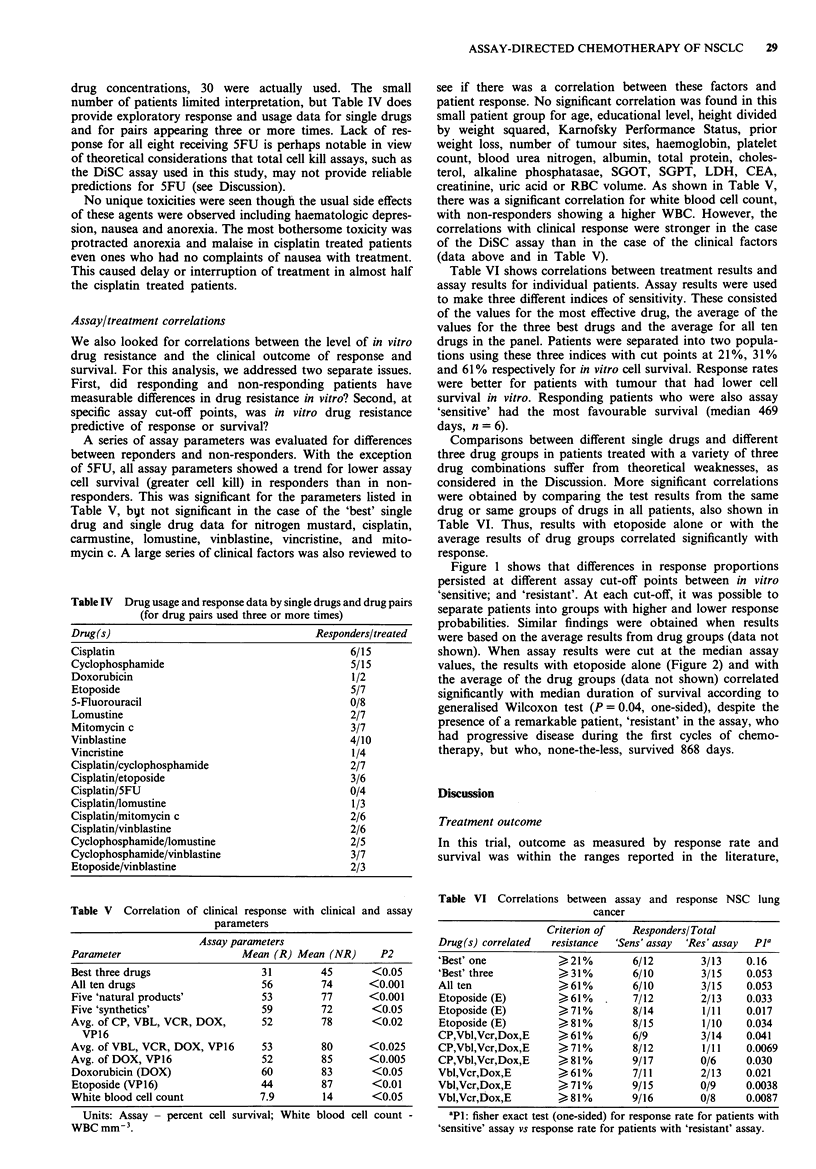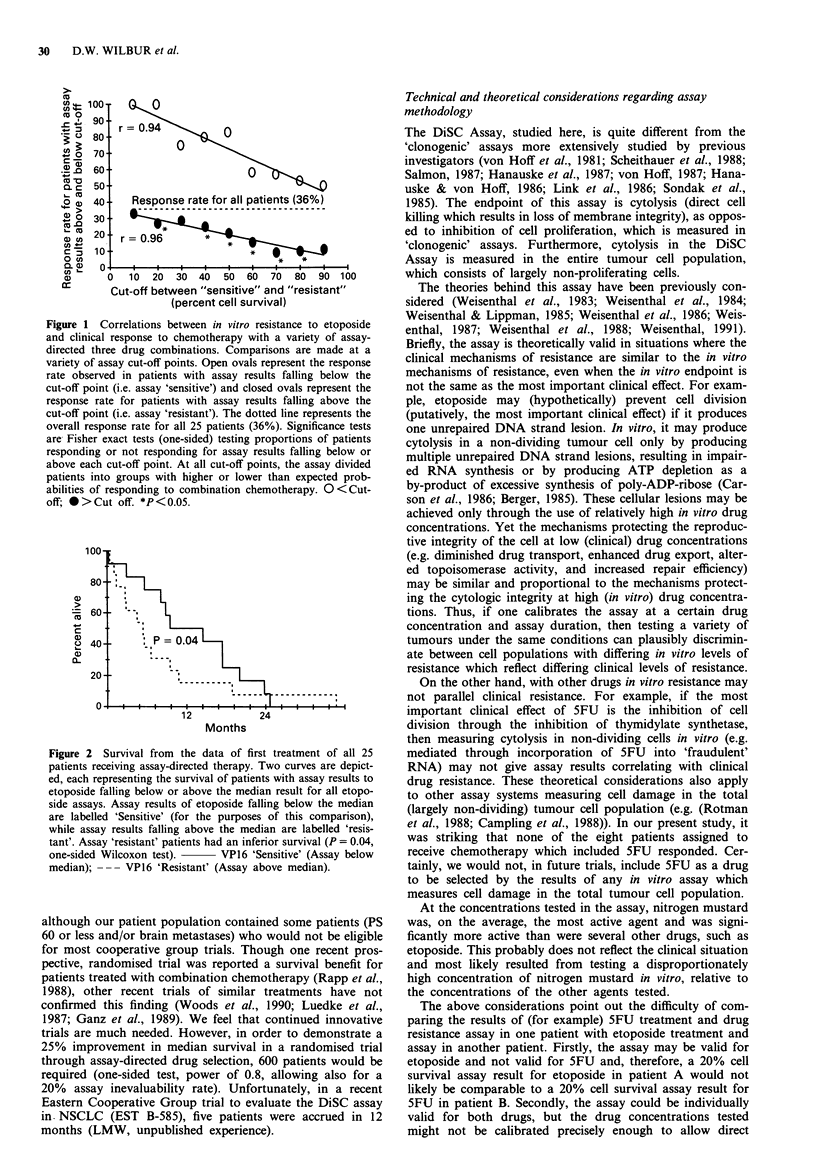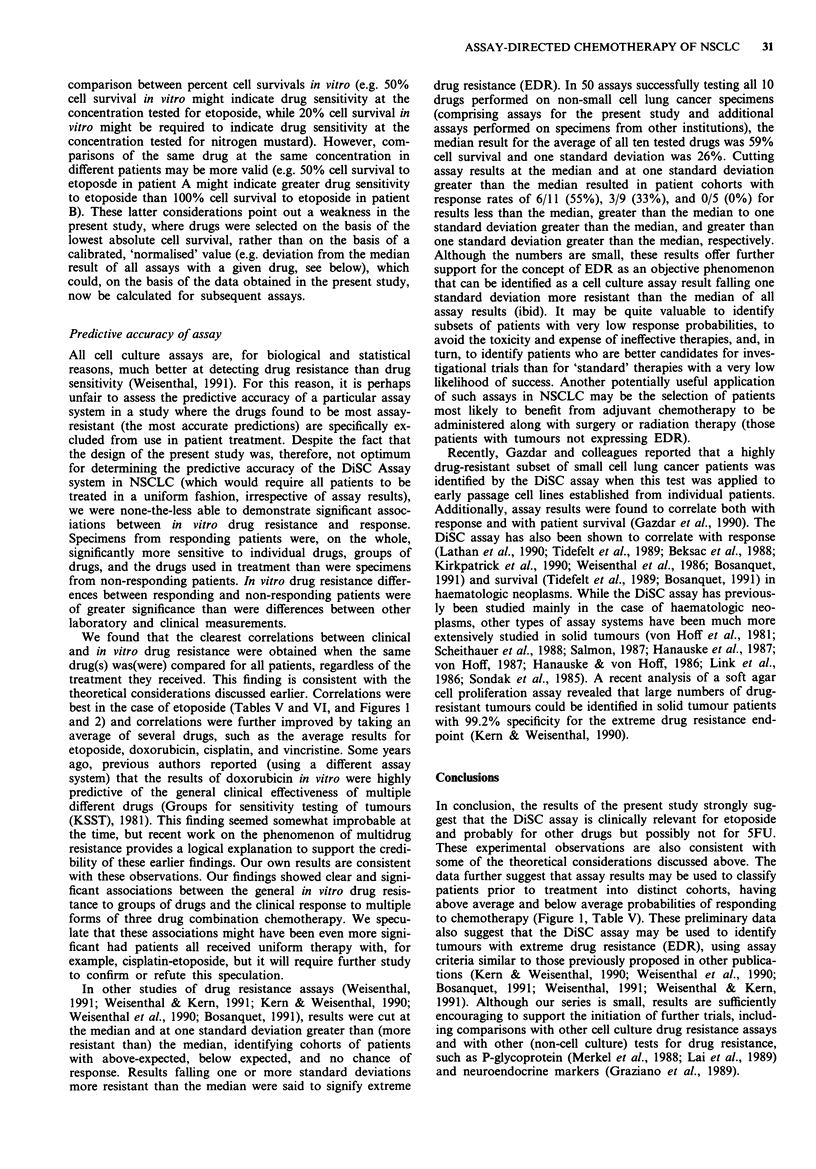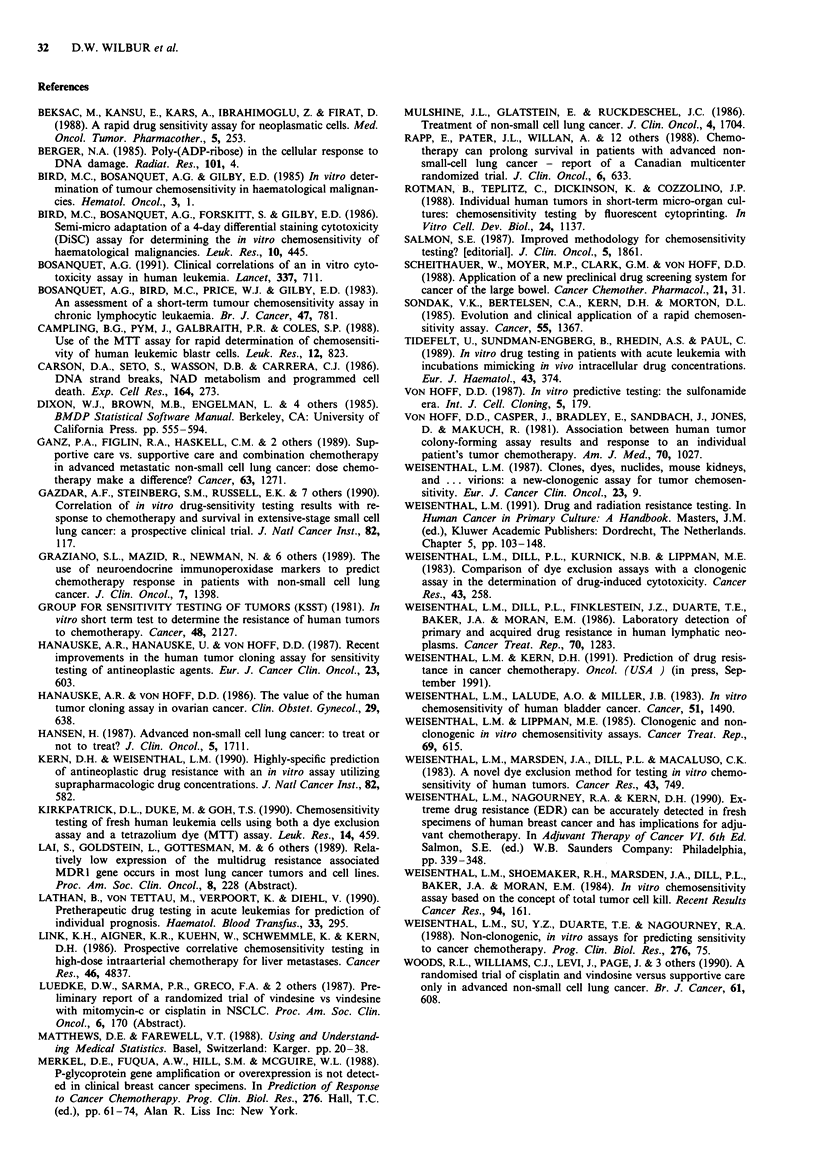# Chemotherapy of non-small cell lung carcinoma guided by an in vitro drug resistance assay measuring total tumour cell kill.

**DOI:** 10.1038/bjc.1992.5

**Published:** 1992-01

**Authors:** D. W. Wilbur, E. S. Camacho, D. A. Hilliard, P. L. Dill, L. M. Weisenthal

**Affiliations:** Pettis Memorial Veterans Hospital, Loma Linda, CA 92357.

## Abstract

Specimens from 45 patients with previously-untreated non-small cell lung cancer (NSCLC) were tested for in vitro chemosensitivity to ten drugs utilising the DiSC assay, which measures cell kill in the total (largely non-dividing) tumour cell population. Thirty-five assays were successful and 25 patients with advanced disease subsequently received chemotherapy with the 'best' three drugs selected by the assay. Six patients were Karnofsky performance status 60 or less and the median pretreatment weight loss was 8.5%. Nine patients had a partial response (response rate = 36%; 95% confidence interval = 17-55%) and the median survival of all patients was 202 days. Specimens from responding patients were significantly more sensitive in the assay to drugs in general (especially to etoposide and to 'natural product' drugs) and to the drugs used in treatment than were specimens from non-responding patients. In vitro drug resistance differences between responding and non-responding patients were of greater significance than were differences between other clinical and laboratory measurements. Assay results classified patients into two cohorts, having relatively high and low probabilities of responding to chemotherapy. Assay results also identified patient cohorts with above average and below average durations of survival. Five patients (20%) were found to have tumours with extreme drug resistance (EDR), defined as assay results for the average of all ten tested drugs falling greater than one standard deviation more resistant than the median for all tumours assayed, and none of these patients with EDR responded to chemotherapy.


*Br. J. Cancer* (1992), **65**, 27–32 © Macmillan Press Ltd., 1992

### Chemotherapy of non-small cell lung carcinoma guided by an *in vitro* drug resistance assay measuring total tumour cell kill

D.W. Wilbur[1,2], E.S. Camacho[1,2], D.A. Hilliard[1], P.L. Dill[3,*] & L.M. Weisenthal[3,4,*]

[1]*Pettis Memorial Veterans Hospital, Loma Linda;* [2]*Loma Linda University School of Medicine;* [3]*Veterans Affairs Medicine Center, Long Beach, and* [4]*University of California, Irvine, California, USA.*

**Summary** Specimens from 45 patients with previously-untreated non-small cell lung cancer (NSCLC) were tested for *in vitro* chemosensitivity to ten drugs utilising the DiSC assay, which measures cell kill in the total (largely non-dividing) tumour cell population. Thirty-five assays were successful and 25 patients with advanced disease subsequently received chemotherapy with the 'best' three drugs selected by the assay. Six patients were Karnofsky performance status 60 or less and the median pretreatment weight loss was 8.5%. Nine patients had a partial response (response rate = 36%; 95% confidence interval = 17–55%) and the median survival of all patients was 202 days. Specimens from responding patients were significantly more sensitive in the assay to drugs in general (especially to etoposide and to 'natural product' drugs) and to the drugs used in treatment than were specimens from non-responding patients. *In vitro* drug resistance differences between responding and non-responding patients were of greater significance than were differences between other clinical and laboratory measurements. Assay results classified patients into two cohorts, having relatively high and low probabilities of responding to chemotherapy. Assay results also identified patient cohorts with above average and below average durations of survival. Five patients (20%) were found to have tumours with extreme drug resistance (EDR), defined as assay results for the average of all ten tested drugs falling greater than one standard deviation more resistant than the median for all tumours assayed, and none of these patients with EDR responded to chemotherapy.

No single program of chemotherapy has emerged as the standard of treatment for patients with advanced non-small cell lung cancer (NSCLC). Randomised comparisons have covered a ranged of options including multidrug and single drug therapy and no chemotherapy (Mulshine *et al.*, 1986; Hansen, 1987). Therapeutic choice has often been without significant impact on survival. Where statistically significant survival improvement has been reported the magnitude of the benefit has been modest and caution seems appropriate in weighing this benefit *vs* the toxicity from the treatment.

One hope for improving treatment outcome has been the provision of customised treatment on the basis of individual tumour properties. From December 1983 through August 1986 we investigated this approach with a pilot study of individualised chemotherapy for NSCLC selected on the basis of an *in vitro* drug resistance assay. The assay chosen was a dye exclusion assay (the DiSc Assay) which has received extensive study in haematologic neoplasms (Weisenthal *et al.*, 1984; Weisenthal *et al.*, 1986; Bird *et al.*, 1985; Bosanquet *et al.*, 1983; Bird *et al.*, 1986; Tidefelt *et al.*, 1989; Kirkpatrick *et al.*, 1990; Lathan *et al.*, 1990; Bosanquet, 1991), but more preliminary evaluation in solid tumours (Weisenthal *et al.*, 1983; Gazdar *et al.*, 1990).

We are now reporting the final results of this trial.

### Methods and materials

### *Patients*

All patients with unresectable NSCLC cared for at the Pettis Memorial Veterans Hospital were potentially eligible if a tumour sample could be obtained without major surgery or incidentally during an otherwise indicated major surgery. Exclusion criteria were failure to obtain a successful *in vitro* assay, a Karnofsky performance score $<40$, a clinical life expectance $<1$ month, a serum creatinine $>2$, uncompensated congestive heart failure or hepatic encephalopathy. Some patients having a potentially curative resection had an *in vitro* study performed on the primary resection specimen. The initial assay results were then available to arrange treatment for a later recurrence. The protocol was approved by the research and human studies committees of the hospital. All patients gave written consent before treatment.

### *Tumour specimens*

Sterile solid tumour specimens were placed directly in lactated Ringers solution (without 5% dextrose) and taken to a laboratory in the same building for mincing and placement in RPMI-1640 with digesting enzymes. Effusions were collected in a heparinised container and if greater than 50 ml were concentrated and reconstituted in 50 ml of autologous fluid. Specimens were sent by (generally overnight) mail from Loma Linda, CA to the laboratory in Long Beach, CA where two of the authors (LMW and PLD) performed the assay.

### *Assay methodology and drug selection*

Previously published methods were used for the assay (Weisenthal *et al.*, 1983; Weisenthal *et al.*, 1986) of the antitumour activity of a panel of ten drugs (nitrogen mustard, cisplatin, lomustine, carmustine, 5-fluorouracil, doxorubicin, vinblastine, vincristine, etoposide and mitomycin C). The DiSC Assay was originally developed to test hematologic neoplasms which grow poorly in culture. Cells are isolated from lymph nodes, effusions, or solid tumour biopsies and cultured in liquid medium in small, polypropylene culture tubes.

Polypropylene is a slippery material which inhibits the attachment and growth of normal cells. After 4–6 days in culture with and without drugs, Fast Green dye is added to the cultures. This dye penetrates the incompetent membranes of dead or dying cells and intensely stains the proteins of the dead or dying cells. Thirty thousand, acetaldehyde-fixed duck red blood cells (DRBC) are added to each culture as an internal standard and the entire cell culture is cytocentrifuged onto a microscope slide. At this point 'living' cells, which have excluded the Fast Green dye, appear clear and unstained, while 'dead' cells and the DRBC stain bright green. Slides are then counterstained with either haematoxylin-eosin or with Wright-Giemsa, which stains the living cells so that

*Present address: Oncotech, Inc., 1791 Kaiser Ave, Irvine, CA 92714, USA.
Correspondence: D.W. Wilbur, The Pettis Memorial Veterans Affairs Medical Center, Loma Linda, CA 92357, USA.
This study was supported by the Department of Veterans Affairs.
Received 6 July 1990; and in revised form 16 August 1991.

they can be identified as tumour cells or as normal cells. A skilled technologist then counts cells to determine the ratio of 'living' tumour cells over DRBC. A ratio of 1:1 in the control cultures would indicate 30,000 'living' tumour cells, and a ratio of 0.1:1 in the drug treated cultures would indicate 3,000 'living' tumour cells, or 10% cell survival, relative to control cultures. Drug concentrations were derived from training set experiments in which concentrations were sought which provided a scattered distribution of results when many different specimens were tested. From the ten drug panel, the three drugs giving the lowest tumour cell survival in the assay were chosen for treatment using the scheme in Table I. Although both carmustine and lomustine were assayed, lomustine was the only nitrosourea considered for use in treatment. Thus, values for carmustine were ignored for the purpose of drug selection but are included in the analysis of assay/treatment correlations, as described in the Results.

### *Feasibility analysis*

Assays were attempted on 45 patients with previously-untreated NSCLC. Thirty-five assays were successful and 25 patients ultimately received protocol-assigned treatment. Assays were attempted on almost all patients (none refused) who were protocol eligible. These 45 patients came from the population of 180 consultations provided for all reasons for non-small cell lung cancer patients during this interval. Of the 20 patients not treated on protocol, four received chemotherapy and none responded. The remaining patients received 'definitive' surgery and/or radiation therapy and/or supportive care.

### *Patient evaluation and statistical methods*

All patients treated with even one dose of chemotherapy were evaluated for response and survival. The customary definitions were used for partial remission (PR) (as at least a 50% decrease in measurable tumour) and for complete remission (CR) (as a disappearance of all known tumour). Bidimensional tumour measurements on chest X-rays and subcutaneous tumour nodules were the most common bases for assessing response. All cases were reviewed by the first author, with confirmation from his colleagues if the judgement was not obvious. Laboratory tests such as the LDH were reviewed to confirm responses assessed by tumour measurements.

Survival curves were calculated by the product limit method and the BMDP statistical package was used for both single factor survival comparisons and multivariate proportional hazards modeling of the survival distribution (Dixon *et al.*, 1985). The Fisher exact test was used for comparisons of response and survival proportions (Matthews & Farewell, 1988). Differences between sample means were compared by T test. Tests were either one- or two-tailed, as noted in the results.

**Table I** Clinical treatment scheme and *in vitro* drug concentrations ($\mu g\,ml^{-1}$) used for testing ('C' = continuous, 4 day drug exposure; '1 h' = 1 h drug exposure)

| *Drug* | *In vitro concentration* | *Clinical scheme 4 week cycles* |
|---|---|---|
| Cisplatin | 1.65°C | 60 mg m$^{-2}$ dl (also d8 first 2) |
| Cyclophosphamide[a] | 1 h | 500 mg m$^{-2}$ dl, d8 |
| Doxorubicin | 1.2 1 h | 25 mg m$^{-2}$ dl, d8 |
| Etoposide | 41.7°C | 100 mg m$^{-2}$ dl, d3, d5 |
| Fluorouracil | 20.0°C | 600 mg m$^{-2}$ dl, d8 |
| Lomustine | 11.0 1 h | 60 mg m$^{-2}$ dl (q 8 wk after 3) |
| Mitomycin C | 0.3 °C | 10 mg m$^{-2}$ dl (q 8 wk) |
| Vinblastine | 0.55°C | 5 mg m$^{-2}$ dl, d8 |
| Vincristine | 0.25°C | 1 mg m$^{-2}$ dl, d8 |

[a]Nitrogen mustard (3.5 $\mu g\,ml^{-1}$) was the assay surrogate for cyclophosphamide. The three drugs giving the lowest *in vitro* tumour survival were combined according to the treatment schedule listed below.

### Results

### *Treatment results*

Some characteristics of the 25 patients at the time of treatment initiation are summarised in Table II. Twenty-three of the patients had some evidence of tumour beyond the mediastinum and the other two had bulky mediastinal tumour. Many of our patients were in poor general condition as shown by the fact that 12 of 25 had albumin levels less than 3.5. Six patients had a Karnofsky performance score of 60 or less. The median pretreatment weight loss was 8.5%. Tumour samples came largely from lymph nodes (mediastinal-4, supraclavicular-8, peripheral-3) but came also from the primary (4) and skin/subcutaneous sites (4). The time from collecting the sample for *in vitro* study to the initiation of chemotherapy was 6 to 439 days but was $<32$ days in 21 of the 25 patients. In only two cases were results from less than the full panel of nine drugs used.

Early deaths (2 in $<21$ days) or failures to return for follow up (1) were considered treatment failures. Table III gives data on response frequency and duration. A 36% objective PR rate was seen with a median survival of 202 days and a median time to progression of 118 days. Although it does not prove a therapeutic benefit, it is of some interest that the responders lived longer than the non-responders ($P = 0.005$, one sided). This at least suggests some biologic differences between these sets of patients. A univariate analysis of clinical data at optimally-selected cut-points revealed a positive correlation of a number of factors with survival, including: WBC less than 9,000 ($P = 0.02$, two-sided), Karnofsky performance status (KPS) greater than 80 ($P = 0.01$, one-sided), height/weight squared greater than 22 ($P = 0.01$, one-sided), and weight loss less than 10.1% ($P = 0.02$, one-sided). Other independent variables not correlated significantly with survival included albumin, LDH, alkaline phosphatase, haemoglobin, and platelet count. Although the 15 patients receiving cisplatin had an insignificantly higher response rate than the patients not receiving cisplatin, survival for the cisplatin-treated patients was shorter (median 141 days for cisplatin-treated patients and 219 days for non-cisplatin-treated patients).

Twenty different drug combinations (of a possible 504) were used, none more than twice. Of a possible 36 pair-wise

**Table II** Patients characteristics at entry onto treatment protocol

| *Characteristics* | | *Patients* |
|---|---|---|
| Number entered | | 25 |
| Age | Mean | 64 yrs |
| | Range | 41–78 yrs |
| Median weight loss | | 8.5% |
| Prior complete resection | | 3 |
| Prior radiation therapy | | 8 |
| Histology | Squamous | 4 |
| | Large cell | 7 |
| | Adeno | 13 |
| LDH elevated | | 8 |
| Alkaline phosphate elevated | | 14 |
| Albumin $<3.5$ | | 12 |
| Performance score (Karnofsky $\leqslant 60$) | | 6 |

**Table III** Response related data

| | | |
|---|---|---|
| *Partial responses*[a] | *All patients* | 9/25 (36%) |
| | *Cisplatin* | 6/15 (40%) |
| | *No cisplatin* | 3/10 (30%) |
| *Median survival*[b] | *All patients* | 202 Days |
| | *Responders* | 369 |
| | *Non-responders* | 125 |
| *Median time to progression*[c] | *All patients* | 118 Days |
| | *Responders* | 195 |
| | *Non-responders* | 46 |

[a]P2 cisplatin *vs* no cisplatin = 0.69; [b]P1 responders *vs* non-responders = 0.005; [c]P1 responders *vs* non-responders = 0.0005.

drug concentrations, 30 were actually used. The small number of patients limited interpretation, but Table IV does provide exploratory response and usage data for single drugs and for pairs appearing three or more times. Lack of response for all eight receiving 5FU is perhaps notable in view of theoretical considerations that total cell kill assays, such as the DiSC assay used in this study, may not provide reliable predictions for 5FU (see Discussion).

No unique toxicities were seen though the usual side effects of these agents were observed including haematologic depression, nausea and anorexia. The most bothersome toxicity was protracted anorexia and malaise in cisplatin treated patients even ones who had no complaints of nausea with treatment. This caused delay or interruption of treatment in almost half the cisplatin treated patients.

*Assay/treatment correlations*

We also looked for correlations between the level of *in vitro* drug resistance and the clinical outcome of response and survival. For this analysis, we addressed two separate issues. First, did responding and non-responding patients have measurable differences in drug resistance *in vitro*? Second, at specific assay cut-off points, was *in vitro* drug resistance predictive of response or survival?

A series of assay parameters was evaluated for differences between reponders and non-responders. With the exception of 5FU, all assay parameters showed a trend for lower assay cell survival (greater cell kill) in responders than in non-responders. This was significant for the parameters listed in Table V, but not significant in the case of the 'best' single drug and single drug data for nitrogen mustard, cisplatin, carmustine, lomustine, vinblastine, vincristine, and mitomycin c. A large series of clinical factors was also reviewed to see if there was a correlation between these factors and patient response. No significant correlation was found in this small patient group for age, educational level, height divided by weight squared, Karnofsky Performance Status, prior weight loss, number of tumour sites, haemoglobin, platelet count, blood urea nitrogen, albumin, total protein, cholesterol, alkaline phosphatasae, SGOT, SGPT, LDH, CEA, creatinine, uric acid or RBC volume. As shown in Table V, there was a significant correlation for white blood cell count, with non-responders showing a higher WBC. However, the correlations with clinical response were stronger in the case of the DiSC assay than in the case of the clinical factors (data above and in Table V).

Table VI shows correlations between treatment results and assay results for individual patients. Assay results were used to make three different indices of sensitivity. These consisted of the values for the most effective drug, the average of the values for the three best drugs and the average for all ten drugs in the panel. Patients were separated into two populations using these three indices with cut points at 21%, 31% and 61% respectively for *in vitro* cell survival. Response rates were better for patients with tumour that had lower cell survival *in vitro*. Responding patients who were also assay 'sensitive' had the most favourable survival (median 469 days, $n = 6$).

Comparisons between different single drugs and different three drug groups in patients treated with a variety of three drug combinations suffer from theoretical weaknesses, as considered in the Discussion. More significant correlations were obtained by comparing the test results from the same drug or same groups of drugs in all patients, also shown in Table VI. Thus, results with etoposide alone or with the average results of drug groups correlated significantly with response.

Figure 1 shows that differences in response proportions persisted at different assay cut-off points between *in vitro* 'sensitive; and 'resistant'. At each cut-off, it was possible to separate patients into groups with higher and lower response probabilities. Similar findings were obtained when results were based on the average results from drug groups (data not shown). When assay results were cut at the median assay values, the results with etoposide alone (Figure 2) and with the average of the drug groups (data not shown) correlated significantly with median duration of survival according to generalised Wilcoxon test ($P = 0.04$, one-sided), despite the presence of a remarkable patient, 'resistant' in the assay, who had progressive disease during the first cycles of chemotherapy, but who, none-the-less, survived 868 days.

**Table IV** Drug usage and response data by single drugs and drug pairs (for drug pairs used three or more times)

| *Drug(s)* | *Responders/treated* |
|---|---|
| Cisplatin | 6/15 |
| Cyclophosphamide | 5/15 |
| Doxorubicin | 1/2 |
| Etoposide | 5/7 |
| 5-Fluorouracil | 0/8 |
| Lomustine | 2/7 |
| Mitomycin c | 3/7 |
| Vinblastine | 4/10 |
| Vincristine | 1/4 |
| Cisplatin/cyclophosphamide | 2/7 |
| Cisplatin/etoposide | 3/6 |
| Cisplatin/5FU | 0/4 |
| Cisplatin/lomustine | 1/3 |
| Cisplatin/mitomycin c | 2/6 |
| Cisplatin/vinblastine | 2/6 |
| Cyclophosphamide/lomustine | 2/5 |
| Cyclophosphamide/vinblastine | 3/7 |
| Etoposide/vinblastine | 2/3 |

**Table V** Correlation of clinical response with clinical and assay parameters

| *Parameter* | *Assay parameters* *Mean (R)* | *Mean (NR)* | *P2* |
|---|---|---|---|
| Best three drugs | 31 | 45 | <0.05 |
| All ten drugs | 56 | 74 | <0.001 |
| Five 'natural products' | 53 | 77 | <0.001 |
| Five 'synthetics' | 59 | 72 | <0.05 |
| Avg. of CP, VBL, VCR, DOX, VP16 | 52 | 78 | <0.02 |
| Avg. of VBL, VCR, DOX, VP16 | 53 | 80 | <0.025 |
| Avg. of DOX, VP16 | 52 | 85 | <0.005 |
| Doxorubicin (DOX) | 60 | 83 | <0.05 |
| Etoposide (VP16) | 44 | 87 | <0.01 |
| White blood cell count | 7.9 | 14 | <0.05 |

Units: Assay – percent cell survival; White blood cell count - WBC $mm^{-3}$.

### Discussion

*Treatment outcome*

In this trial, outcome as measured by response rate and survival was within the ranges reported in the literature,

**Table VI** Correlations between assay and response NSC lung cancer

| *Drug(s) correlated* | *Criterion of resistance* | *Responders/Total* *'Sens' assay* | *'Res' assay* | *P1*[a] |
|---|---|---|---|---|
| 'Best' one | ≥21% | 6/12 | 3/13 | 0.16 |
| 'Best' three | ≥31% | 6/10 | 3/15 | 0.053 |
| All ten | ≥61% | 6/10 | 3/15 | 0.053 |
| Etoposide (E) | ≥61% | 7/12 | 2/13 | 0.033 |
| Etoposide (E) | ≥71% | 8/14 | 1/11 | 0.017 |
| Etoposide (E) | ≥81% | 8/15 | 1/10 | 0.034 |
| CP,Vbl,Vcr,Dox,E | ≥61% | 6/9 | 3/14 | 0.041 |
| CP,Vbl,Vcr,Dox,E | ≥71% | 8/12 | 1/11 | 0.0069 |
| CP,Vbl,Vcr,Dox,E | ≥81% | 9/17 | 0/6 | 0.030 |
| Vbl,Vcr,Dox,E | ≥61% | 7/11 | 2/13 | 0.021 |
| Vbl,Vcr,Dox,E | ≥71% | 9/15 | 0/9 | 0.0038 |
| Vbl,Vcr,Dox,E | ≥81% | 9/16 | 0/8 | 0.0087 |

[a]P1: fisher exact test (one-sided) for response rate for patients with 'sensitive' assay *vs* response rate for patients with 'resistant' assay.

**Figure 1** Correlations between *in vitro* resistance to etoposide and clinical response to chemotherapy with a variety of assay-directed three drug combinations. Comparisons are made at a variety of assay cut-off points. Open ovals represent the response rate observed in patients with assay results falling below the cut-off point (i.e. assay 'sensitive') and closed ovals represent the response rate for patients with assay results falling above the cut-off point (i.e. assay 'resistant'). The dotted line represents the overall response rate for all 25 patients (36%). Significance tests are Fisher exact tests (one-sided) testing proportions of patients responding or not responding for assay results falling below or above each cut-off point. At all cut-off points, the assay divided patients into groups with higher or lower than expected probabilities of responding to combination chemotherapy. O<Cut-off; ●>Cut off. $^*P<0.05$.

**Figure 2** Survival from the data of first treatment of all 25 patients receiving assay-directed therapy. Two curves are depicted, each representing the survival of patients with assay results to etoposide falling below or above the median result for all etoposide assays. Assay results of etoposide falling below the median are labelled 'Sensitive' (for the purposes of this comparison), while assay results falling above the median are labelled 'resistant'. Assay 'resistant' patients had an inferior survival ($P = 0.04$, one-sided Wilcoxon test). ——— VP16 'Sensitive' (Assay below median); - - - VP16 'Resistant' (Assay above median).

although our patient population contained some patients (PS 60 or less and/or brain metastases) who would not be eligible for most cooperative group trials. Though one recent prospective, randomised trial was reported a survival benefit for patients treated with combination chemotherapy (Rapp *et al.*, 1988), other recent trials of similar treatments have not confirmed this finding (Woods *et al.*, 1990; Luedke *et al.*, 1987; Ganz *et al.*, 1989). We feel that continued innovative trials are much needed. However, in order to demonstrate a 25% improvement in median survival in a randomised trial through assay-directed drug selection, 600 patients would be required (one-sided test, power of 0.8, allowing also for a 20% assay inevaluability rate). Unfortunately, in a recent Eastern Cooperative Group trial to evaluate the DiSC assay in NSCLC (EST B-585), five patients were accrued in 12 months (LMW, unpublished experience).

*Technical and theoretical considerations regarding assay methodology*

The DiSC Assay, studied here, is quite different from the 'clonogenic' assays more extensively studied by previous investigators (von Hoff *et al.*, 1981; Scheithauer *et al.*, 1988; Salmon, 1987; Hanauske *et al.*, 1987; von Hoff, 1987; Hanauske & von Hoff, 1986; Link *et al.*, 1986; Sondak *et al.*, 1985). The endpoint of this assay is cytolysis (direct cell killing which results in loss of membrane integrity), as opposed to inhibition of cell proliferation, which is measured in 'clonogenic' assays. Furthermore, cytolysis in the DiSC Assay is measured in the entire tumour cell population, which consists of largely non-proliferating cells.

The theories behind this assay have been previously considered (Weisenthal *et al.*, 1983; Weisenthal *et al.*, 1984; Weisenthal & Lippman, 1985; Weisenthal *et al.*, 1986; Weisenthal, 1987; Weisenthal *et al.*, 1988; Weisenthal, 1991). Briefly, the assay is theoretically valid in situations where the clinical mechanisms of resistance are similar to the *in vitro* mechanisms of resistance, even when the *in vitro* endpoint is not the same as the most important clinical effect. For example, etoposide may (hypothetically) prevent cell division (putatively, the most important clinical effect) if it produces one unrepaired DNA strand lesion. *In vitro*, it may produce cytolysis in a non-dividing tumour cell only by producing multiple unrepaired DNA strand lesions, resulting in impaired RNA synthesis or by producing ATP depletion as a by-product of excessive synthesis of poly-ADP-ribose (Carson *et al.*, 1986; Berger, 1985). These cellular lesions may be achieved only through the use of relatively high *in vitro* drug concentrations. Yet the mechanisms protecting the reproductive integrity of the cell at low (clinical) drug concentrations (e.g. diminished drug transport, enhanced drug export, altered topoisomerase activity, and increased repair efficiency) may be similar and proportional to the mechanisms protecting the cytologic integrity at high (*in vitro*) drug concentrations. Thus, if one calibrates the assay at a certain drug concentration and assay duration, then testing a variety of tumours under the same conditions can plausibly discriminate between cell populations with differing *in vitro* levels of resistance which reflect differing clinical levels of resistance.

On the other hand, with other drugs *in vitro* resistance may not parallel clinical resistance. For example, if the most important clinical effect of 5FU is the inhibition of cell division through the inhibition of thymidylate synthetase, then measuring cytolysis in non-dividing cells *in vitro* (e.g. mediated through incorporation of 5FU into 'fraudulent' RNA) may not give assay results correlating with clinical drug resistance. These theoretical considerations also apply to other assay systems measuring cell damage in the total (largely non-dividing) tumour cell population (e.g. (Rotman *et al.*, 1988; Campling *et al.*, 1988)). In our present study, it was striking that none of the eight patients assigned to receive chemotherapy which included 5FU responded. Certainly, we would not, in future trials, include 5FU as a drug to be selected by the results of any *in vitro* assay which measures cell damage in the total tumour cell population.

At the concentrations tested in the assay, nitrogen mustard was, on the average, the most active agent and was significantly more active than were several other drugs, such as etoposide. This probably does not reflect the clinical situation and most likely resulted from testing a disproportionately high concentration of nitrogen mustard *in vitro*, relative to the concentrations of the other agents tested.

The above considerations point out the difficulty of comparing the results of (for example) 5FU treatment and drug resistance assay in one patient with etoposide treatment and assay in another patient. Firstly, the assay may be valid for etoposide and not valid for 5FU and, therefore, a 20% cell survival assay result for etoposide in patient A would not likely be comparable to a 20% cell survival assay result for 5FU in patient B. Secondly, the assay could be individually valid for both drugs, but the drug concentrations tested might not be calibrated precisely enough to allow direct comparison between percent cell survivals *in vitro* (e.g. 50% cell survival *in vitro* might indicate drug sensitivity at the concentration tested for etoposide, while 20% cell survival *in vitro* might be required to indicate drug sensitivity at the concentration tested for nitrogen mustard). However, comparisons of the same drug at the same concentration in different patients may be more valid (e.g. 50% cell survival to etoposde in patient A might indicate greater drug sensitivity to etoposide than 100% cell survival to etoposide in patient B). These latter considerations point out a weakness in the present study, where drugs were selected on the basis of the lowest absolute cell survival, rather than on the basis of a calibrated, 'normalised' value (e.g. deviation from the median result of all assays with a given drug, see below), which could, on the basis of the data obtained in the present study, now be calculated for subsequent assays.

*Predictive accuracy of assay*

All cell culture assays are, for biological and statistical reasons, much better at detecting drug resistance than drug sensitivity (Weisenthal, 1991). For this reason, it is perhaps unfair to assess the predictive accuracy of a particular assay system in a study where the drugs found to be most assay-resistant (the most accurate predictions) are specifically excluded from use in patient treatment. Despite the fact that the design of the present study was, therefore, not optimum for determining the predictive accuracy of the DiSC Assay system in NSCLC (which would require all patients to be treated in a uniform fashion, irrespective of assay results), we were none-the-less able to demonstrate significant associations between *in vitro* drug resistance and response. Specimens from responding patients were, on the whole, significantly more sensitive to individual drugs, groups of drugs, and the drugs used in treatment than were specimens from non-responding patients. *In vitro* drug resistance differences between responding and non-responding patients were of greater significance than were differences between other laboratory and clinical measurements.

We found that the clearest correlations between clinical and *in vitro* drug resistance were obtained when the same drug(s) was(were) compared for all patients, regardless of the treatment they received. This finding is consistent with the theoretical considerations discussed earlier. Correlations were best in the case of etoposide (Tables V and VI, and Figures 1 and 2) and correlations were further improved by taking an average of several drugs, such as the average results for etoposide, doxorubicin, cisplatin, and vincristine. Some years ago, previous authors reported (using a different assay system) that the results of doxorubicin *in vitro* were highly predictive of the general clinical effectiveness of multiple different drugs (Groups for sensitivity testing of tumours (KSST), 1981). This finding seemed somewhat improbable at the time, but recent work on the phenomenon of multidrug resistance provides a logical explanation to support the credibility of these earlier findings. Our own results are consistent with these observations. Our findings showed clear and significant associations between the general *in vitro* drug resistance to groups of drugs and the clinical response to multiple forms of three drug combination chemotherapy. We speculate that these associations might have been even more significant had patients all received uniform therapy with, for example, cisplatin-etoposide, but it will require further study to confirm or refute this speculation.

In other studies of drug resistance assays (Weisenthal, 1991; Weisenthal & Kern, 1991; Kern & Weisenthal, 1990; Weisenthal *et al.*, 1990; Bosanquet, 1991), results were cut at the median and at one standard deviation greater than (more resistant than) the median, identifying cohorts of patients with above-expected, below expected, and no chance of response. Results falling one or more standard deviations more resistant than the median were said to signify extreme drug resistance (EDR). In 50 assays successfully testing all 10 drugs performed on non-small cell lung cancer specimens (comprising assays for the present study and additional assays performed on specimens from other institutions), the median result for the average of all ten tested drugs was 59% cell survival and one standard deviation was 26%. Cutting assay results at the median and at one standard deviation greater than the median resulted in patient cohorts with response rates of 6/11 (55%), 3/9 (33%), and 0/5 (0%) for results less than the median, greater than the median to one standard deviation greater than the median, and greater than one standard deviation greater than the median, respectively. Although the numbers are small, these results offer further support for the concept of EDR as an objective phenomenon that can be identified as a cell culture assay result falling one standard deviation more resistant than the median of all assay results (ibid). It may be quite valuable to identify subsets of patients with very low response probabilities, to avoid the toxicity and expense of ineffective therapies, and, in turn, to identify patients who are better candidates for investigational trials than for 'standard' therapies with a very low likelihood of success. Another potentially useful application of such assays in NSCLC may be the selection of patients most likely to benefit from adjuvant chemotherapy to be administered along with surgery or radiation therapy (those patients with tumours not expressing EDR).

Recently, Gazdar and colleagues reported that a highly drug-resistant subset of small cell lung cancer patients was identified by the DiSC assay when this test was applied to early passage cell lines established from individual patients. Additionally, assay results were found to correlate both with response and with patient survival (Gazdar *et al.*, 1990). The DiSC assay has also been shown to correlate with response (Lathan *et al.*, 1990; Tidefelt *et al.*, 1989; Beksac *et al.*, 1988; Kirkpatrick *et al.*, 1990; Weisenthal *et al.*, 1986; Bosanquet, 1991) and survival (Tidefelt *et al.*, 1989; Bosanquet, 1991) in haematologic neoplasms. While the DiSC assay has previously been studied mainly in the case of haematologic neoplasms, other types of assay systems have been much more extensively studied in solid tumours (von Hoff *et al.*, 1981; Scheithauer *et al.*, 1988; Salmon, 1987; Hanauske *et al.*, 1987; von Hoff, 1987; Hanauske & von Hoff, 1986; Link *et al.*, 1986; Sondak *et al.*, 1985). A recent analysis of a soft agar cell proliferation assay revealed that large numbers of drug-resistant tumours could be identified in solid tumour patients with 99.2% specificity for the extreme drug resistance end-point (Kern & Weisenthal, 1990).

### Conclusions

In conclusion, the results of the present study strongly suggest that the DiSC assay is clinically relevant for etoposide and probably for other drugs but possibly not for 5FU. These experimental observations are also consistent with some of the theoretical considerations discussed above. The data further suggest that assay results may be used to classify patients prior to treatment into distinct cohorts, having above average and below average probabilities of responding to chemotherapy (Figure 1, Table V). These preliminary data also suggest that the DiSC assay may be used to identify tumours with extreme drug resistance (EDR), using assay criteria similar to those previously proposed in other publications (Kern & Weisenthal, 1990; Weisenthal *et al.*, 1990; Bosanquet, 1991; Weisenthal, 1991; Weisenthal & Kern, 1991). Although our series is small, results are sufficiently encouraging to support the initiation of further trials, including comparisons with other cell culture drug resistance assays and with other (non-cell culture) tests for drug resistance, such as P-glycoprotein (Merkel *et al.*, 1988; Lai *et al.*, 1989) and neuroendocrine markers (Graziano *et al.*, 1989).

### References

BEKSAC, M., KANSU, E., KARS, A., IBRAHIMOGLU, Z. & FIRAT, D. (1988). A rapid drug sensitivity assay for neoplasmatic cells. *Med. Oncol. Tumor. Pharmacother.*, **5**, 253.

BERGER, N.A. (1985). Poly-(ADP-ribose) in the cellular response to DNA damage. *Radiat. Res.*, **101**, 4.

BIRD, M.C., BOSANQUET, A.G. & GILBY, E.D. (1985) *In vitro* determination of tumour chemosensitivity in haematological malignancies. *Hematol. Oncol.*, **3**, 1.

BIRD, M.C., BOSANQUET, A.G., FORSKITT, S. & GILBY, E.D. (1986). Semi-micro adaptation of a 4-day differential staining cytotoxicity (DiSC) assay for determining the *in vitro* chemosensitivity of haematological malignancies. *Leuk. Res.*, **10**, 445.

BOSANQUET, A.G. (1991). Clinical correlations of an in vitro cytotoxicity assay in human leukemia. *Lancet*, **337**, 711.

BOSANQUET, A.G., BIRD, M.C., PRICE, W.J. & GILBY, E.D. (1983). An assessment of a short-term tumour chemosensitivity assay in chronic lymphocytic leukaemia. *Br. J. Cancer*, **47**, 781.

CAMPLING, B.G., PYM, J., GALBRAITH, P.R. & COLES, S.P. (1988). Use of the MTT assay for rapid determination of chemosensitivity of human leukemic blastr cells. *Leuk. Res.*, **12**, 823.

CARSON, D.A., SETO, S., WASSON, D.B. & CARRERA, C.J. (1986). DNA strand breaks, NAD metabolism and programmed cell death. *Exp. Cell Res.*, **164**, 273.

DIXON, W.J., BROWN, M.B., ENGELMAN, L. & 4 others (1985). *BMDP Statistical Software Manual.* Berkeley, CA: University of California Press. pp. 555–594.

GANZ, P.A., FIGLIN, R.A., HASKELL, C.M. & 2 others (1989). Supportive care vs. supportive care and combination chemotherapy in advanced metastatic non-small cell lung cancer: dose chemotherapy make a difference? *Cancer*, **63**, 1271.

GAZDAR, A.F., STEINBERG, S.M., RUSSELL, E.K. & 7 others (1990). Correlation of *in vitro* drug-sensitivity testing results with response to chemotherapy and survival in extensive-stage small cell lung cancer: a prospective clinical trial. *J. Natl Cancer Inst.*, **82**, 117.

GRAZIANO, S.L., MAZID, R., NEWMAN, N. & 6 others (1989). The use of neuroendocrine immunoperoxidase markers to predict chemotherapy response in patients with non-small cell lung cancer. *J. Clin. Oncol.*, **7**, 1398.

GROUP FOR SENSITIVITY TESTING OF TUMORS (KSST) (1981). *In vitro* short term test to determine the resistance of human tumors to chemotherapy. *Cancer*, **48**, 2127.

HANAUSKE, A.R., HANAUSKE, U. & VON HOFF, D.D. (1987). Recent improvements in the human tumor cloning assay for sensitivity testing of antineoplastic agents. *Eur. J. Cancer Clin. Oncol.*, **23**, 603.

HANAUSKE, A.R. & VON HOFF, D.D. (1986). The value of the human tumor cloning assay in ovarian cancer. *Clin. Obstet. Gynecol.*, **29**, 638.

HANSEN, H. (1987). Advanced non-small cell lung cancer: to treat or not to treat? *J. Clin. Oncol.*, **5**, 1711.

KERN, D.H. & WEISENTHAL, L.M. (1990). Highly-specific prediction of antineoplastic drug resistance with an *in vitro* assay utilizing suprapharmacologic drug concentrations. *J. Natl Cancer Inst.*, **82**, 582.

KIRKPATRICK, D.L., DUKE, M. & GOH, T.S. (1990). Chemosensitivity testing of fresh human leukemia cells using both a dye exclusion assay and a tetrazolium dye (MTT) assay. *Leuk. Res.*, **14**, 459.

LAI, S., GOLDSTEIN, L., GOTTESMAN, M. & 6 others (1989). Relatively low expression of the multidrug resistance associated MDR1 gene occurs in most lung cancer tumors and cell lines. *Proc. Am. Soc. Clin. Oncol.*, **8**, 228 (Abstract).

LATHAN, B., VON TETTAU, M., VERPOORT, K. & DIEHL, V. (1990). Pretherapeutic drug testing in acute leukemias for prediction of individual prognosis. *Haematol. Blood Transfus.*, **33**, 295.

LINK, K.H., AIGNER, K.R., KUEHN, W., SCHWEMMLE, K. & KERN, D.H. (1986). Prospective correlative chemosensitivity testing in high-dose intraarterial chemotherapy for liver metastases. *Cancer Res.*, **46**, 4837.

LUEDKE, D.W., SARMA, P.R., GRECO, F.A. & 2 others (1987). Preliminary report of a randomized trial of vindesine vs vindesine with mitomycin-c or cisplatin in NSCLC. *Proc. Am. Soc. Clin. Oncol.*, **6**, 170 (Abstract).

MATTHEWS, D.E. & FAREWELL, V.T. (1988). *Using and Understanding Medical Statistics.* Basel, Switzerland: Karger. pp. 20–38.

MERKEL, D.E., FUQUA, A.W., HILL, S.M. & MCGUIRE, W.L. (1988). P-glycoprotein gene amplification or overexpression is not detected in clinical breast cancer specimens. In *Prediction of Response to Cancer Chemotherapy. Prog. Clin. Biol. Res.*, **276**. Hall, T.C. (ed.), pp. 61–74, Alan R. Liss Inc: New York.

MULSHINE, J.L., GLATSTEIN, E. & RUCKDESCHEL, J.C. (1986). Treatment of non-small cell lung cancer. *J. Clin. Oncol.*, **4**, 1704.

RAPP, E., PATER, J.L., WILLAN, A. & 12 others (1988). Chemotherapy can prolong survival in patients with advanced non-small-cell lung cancer – report of a Canadian multicenter randomized trial. *J. Clin. Oncol.*, **6**, 633.

ROTMAN, B., TEPLITZ, C., DICKINSON, K. & COZZOLINO, J.P. (1988). Individual human tumors in short-term micro-organ cultures: chemosensitivity testing by fluorescent cytoprinting. *In Vitro Cell. Dev. Biol.*, **24**, 1137.

SALMON, S.E. (1987). Improved methodology for chemosensitivity testing? [editorial]. *J. Clin. Oncol.*, **5**, 1861.

SCHEITHAUER, W., MOYER, M.P., CLARK, G.M. & VON HOFF, D.D. (1988). Application of a new preclinical drug screening system for cancer of the large bowel. *Cancer Chemother. Pharmacol.*, **21**, 31.

SONDAK, V.K., BERTELSEN, C.A., KERN, D.H. & MORTON, D.L. (1985). Evolution and clinical application of a rapid chemosensitivity assay. *Cancer*, **55**, 1367.

TIDEFELT, U., SUNDMAN-ENGBERG, B., RHEDIN, A.S. & PAUL, C. (1989). *In vitro* drug testing in patients with acute leukemia with incubations mimicking *in vivo* intracellular drug concentrations. *Eur. J. Haematol.*, **43**, 374.

VON HOFF, D.D. (1987). *In vitro* predictive testing: the sulfonamide era. *Int. J. Cell. Cloning*, **5**, 179.

VON HOFF, D.D., CASPER, J., BRADLEY, E., SANDBACH, J., JONES, D. & MAKUCH, R. (1981). Association between human tumor colony-forming assay results and response to an individual patient's tumor chemotherapy. *Am. J. Med.*, **70**, 1027.

WEISENTHAL, L.M. (1987). Clones, dyes, nuclides, mouse kidneys, and ... virions: a new-clonogenic assay for tumor chemosensitivity. *Eur. J. Cancer Clin. Oncol.*, **23**, 9.

WEISENTHAL, L.M. (1991). Drug and radiation resistance testing. In *Human Cancer in Primary Culture: A Handbook.* Masters, J.M. (ed.), Kluwer Academic Publishers: Dordrecht, The Netherlands. Chapter 5, pp. 103–148.

WEISENTHAL, L.M., DILL, P.L., KURNICK, N.B. & LIPPMAN, M.E. (1983). Comparison of dye exclusion assays with a clonogenic assay in the determination of drug-induced cytotoxicity. *Cancer Res.*, **43**, 258.

WEISENTHAL, L.M., DILL, P.L., FINKLESTEIN, J.Z., DUARTE, T.E., BAKER, J.A. & MORAN, E.M. (1986). Laboratory detection of primary and acquired drug resistance in human lymphatic neoplasms. *Cancer Treat. Rep.*, **70**, 1283.

WEISENTHAL, L.M. & KERN, D.H. (1991). Prediction of drug resistance in cancer chemotherapy. *Oncol. (USA )* (in press, September 1991).

WEISENTHAL, L.M., LALUDE, A.O. & MILLER, J.B. (1983). *In vitro* chemosensitivity of human bladder cancer. *Cancer*, **51**, 1490.

WEISENTHAL, L.M. & LIPPMAN, M.E. (1985). Clonogenic and non-clonogenic *in vitro* chemosensitivity assays. *Cancer Treat. Rep.*, **69**, 615.

WEISENTHAL, L.M., MARSDEN, J.A., DILL, P.L. & MACALUSO, C.K. (1983). A novel dye exclusion method for testing *in vitro* chemosensitivity of human tumors. *Cancer Res.*, **43**, 749.

WEISENTHAL, L.M., NAGOURNEY, R.A. & KERN, D.H. (1990). Extreme drug resistance (EDR) can be accurately detected in fresh specimens of human breast cancer and has implications for adjuvant chemotherapy. In *Adjuvant Therapy of Cancer VI. 6th Ed.* Salmon, S.E. (ed.) W.B. Saunders Company: Philadelphia, pp. 339–348.

WEISENTHAL, L.M., SHOEMAKER, R.H., MARSDEN, J.A., DILL, P.L., BAKER, J.A. & MORAN, E.M. (1984). *In vitro* chemosensitivity assay based on the concept of total tumor cell kill. *Recent Results Cancer Res.*, **94**, 161.

WEISENTHAL, L.M., SU, Y.Z., DUARTE, T.E. & NAGOURNEY, R.A. (1988). Non-clonogenic, *in vitro* assays for predicting sensitivity to cancer chemotherapy. *Prog. Clin. Biol. Res.*, **276**, 75.

WOODS, R.L., WILLIAMS, C.J., LEVI, J., PAGE, J. & 3 others (1990). A randomised trial of cisplatin and vindosine versus supportive care only in advanced non-small cell lung cancer. *Br. J. Cancer*, **61**, 608.